# Study protocol comparing the ethical, psychological and socio-economic impact of personalised breast cancer screening to that of standard screening in the “My Personal Breast Screening” (MyPeBS) randomised clinical trial

**DOI:** 10.1186/s12885-022-09484-6

**Published:** 2022-05-06

**Authors:** Alexandra Roux, Rachel Cholerton, Jonathan Sicsic, Nora Moumjid, David P. French, Paolo Giorgi Rossi, Corinne Balleyguier, Michal Guindy, Fiona J. Gilbert, Jean-Benoit Burrion, Xavier Castells, David Ritchie, Debbie Keatley, Camille Baron, Suzette Delaloge, Sandrine de Montgolfier

**Affiliations:** 1 IRIS (UMR8156 CNRS & U997 INSERM), Paris 13 University, Aubervilliers, France; 2 University of Manchester, Manchester, UK; 3 Université de Paris, LIRAES F-75006, Paris, France; 4 Université Lyon 1, P2S EA 4129, Centre Léon Bérard, F-69373 Lyon, France; 5 AUSL - IRCCS Di Reggio Emilia, Reggio Emilia, Italy; 6 Institut Gustave Roussy, Villejuif, France; 7 Assuta Medical Centers, Tel Aviv, Israel; 8 Ben Gurion University, Beersheba, Israel; 9 University of Cambridge, Cambridge, UK; 10 Institut Jules Bordet, Brussels, Belgium; 11 IMIM (Hospital del Mar Medical Research Institute), Barcelona, Spain; 12 European Cancer Leagues, Brussels, Belgium; 13 Independent Cancer Patients’ Voice, London, UK; 14 Unicancer, Paris, France; 15 Paris Est Creteil University, Créteil, France

**Keywords:** Risk-stratification, Breast cancer screening, Psychological impact, Comprehension, Inequity, Underserved populations, Satisfaction

## Abstract

**Background:**

The MyPeBS study is an ongoing randomised controlled trial testing whether a risk-stratified breast cancer screening strategy is non-inferior, or eventually superior, to standard age-based screening at reducing incidence of stage 2 or more cancers. This large European Commission-funded initiative aims to include 85,000 women aged 40 to 70 years, without prior breast cancer and not previously identified at high risk in six countries (Belgium, France, Italy, Israel, Spain, UK). A specific work package within MyPeBS examines psychological, socio-economic and ethical aspects of this new screening strategy. It compares women’s reported data and outcomes in both trial arms on the following issues: general anxiety, cancer-related worry, understanding of breast cancer screening strategy and information-seeking behaviour, socio-demographic and economic characteristics, quality of life, risk perception, intention to change health-related behaviours, satisfaction with the trial.

**Methods:**

At inclusion, 3-months, 1-year and 4-years, each woman participating in MyPeBS is asked to fill online questionnaires. Descriptive statistics, bivariate analyses, subgroup comparisons and analysis of variations over time will be performed with appropriate tests to assess differences between arms. Multivariate regression models will allow modelling of different patient reported data and outcomes such as comprehension of the information provided, general anxiety or cancer worry, and information seeking behaviour. In addition, a qualitative study (48 semi-structured interviews conducted in France and in the UK with women randomised in the risk-stratified arm), will help further understand participants’ acceptability and comprehension of the trial, and their experience of risk assessment.

**Discussion:**

Beyond the scientific and medical objectives of this clinical study, it is critical to acknowledge the consequences of such a paradigm shift for women. Indeed, introducing a risk-based screening relying on individual biological differences also implies addressing non-biological differences (e.g. social status or health literacy) from an ethical perspective, to ensure equal access to healthcare. The results of the present study will facilitate making recommendations on implementation at the end of the trial to accompany any potential change in screening strategy.

**Trial registration:**

Study sponsor: UNICANCER. My personalised breast screening (MyPeBS). Clinicaltrials.gov (2018) available at: https://clinicaltrials.gov/ct2/show/NCT03672331

Contact: Cécile VISSAC SABATIER, PhD, + 33 (0)1 73 79 77 58 ext + 330,142,114,293, contact@mypebs.eu.

**Supplementary Information:**

The online version contains supplementary material available at 10.1186/s12885-022-09484-6.

## Background

Breast cancer is the leading cause of death among women worldwide [1]. Breast screening programmes use age as the primary criterion for eligibility and primarily target women over 50 years of age.^1^ Across countries, numerous scientific and medical debates exist on the optimal screening interval, and on whether younger women would also benefit from screening programmes. The discussions focus on the need to improve the balance of benefits of mammographic screening relative to potential harms including false-positive and false-negative findings, over diagnosis, over treatment, and the associated anxiety and unnecessary costs [2–4]. Recent reviews of the evidence on breast cancer screening found clear support that screening prevents early deaths, but also made such harms apparent and suggested more flexible strategies may help [5, 6].

Since the risk of developing breast cancer varies widely among women (according to age, genetics, family history, history of benign breast disease, BMI, hormone and other exposures, etc.), risk-based approaches to breast cancer screening have recently emerged as promising cancer prevention strategies [7–9]. These require applying risk assessment to the whole screening population, stratifying the population into several risk groups, assigning individuals to a risk group, and tailoring prevention and early detection interventions to each risk group [10]. Potential benefits of risk stratified screening for women identified as being at higher risk could include increased awareness, more frequent and sensitive screening, and being offered interventions that can reduce their risk [11]. Internationally, there are several ongoing trials analysing these outcomes under risk-stratified screening in comparison to routine breast cancer screening. These include the BC-Predict project in the UK [12], Perspective in Canada [13], and two controlled randomised trial: the Wisdom study in the US [14], and the MyPeBS study (“My Personal Breast Screening”) in Europe and Israel.

The MyPeBS study^2^ is an ongoing randomised controlled trial currently running in six countries (Italy, France, Israel, the UK, Belgium, and Spain) that is testing whether a risk-stratified screening strategy is non-inferior, or eventually better at reducing incidence of advanced breast cancer (i.e. cancers diagnosed at stage 2 or higher), than standard age-based screening. Women from the general population aged 40 to 70 are eligible if they live in a participating area from one of the six participating countries and have never had breast cancer or a high breast cancer risk condition. Participants are randomised 1:1 between the standard arm and the risk-based arm. In the standard arm, women are screened for breast cancer according to the current national guidelines and procedures (bi-yearly or tri-yearly mammogram and/or tomosynthesis, with ultrasound in case of dense breast, starting at age 45–50, up to age 69–74 depending on countries). For the women in the risk-based arm, screening frequency and method depend on their individual 5-year predicted risk of invasive breast cancer, which is estimated at study entry. Risk stratification is done using validated clinical risk scores including a polygenic risk score performed on DNA extracted from a saliva sample and comprising 313 relevant polymorphisms, adjusted for national breast cancer incidence [15]. The primary endpoint (incidence of stage 2 + breast cancer) is measured after four years, at the end of follow-up. Secondary endpoints include false negative and false positive findings, over diagnosis, cost-effectiveness, long-term breast cancer specific mortality, and the present patient-reported data and outcomes.

This study is relevant to address the relative impact of risk-stratified screening for several reasons. First, it is a very large study, with 85,000 women to be randomised. Second, each of the countries in the MyPeBS study have a routine screening programme in which the study is inserted, and against which any additional benefits and harms of risk-stratified screening can be readily compared. Third, it takes place across six different countries with differing screening programmes, healthcare organisations, cultures and ethnic minority populations. Given this, any comparisons of the two approaches to screening will take into account these background factors, to allow the conclusions to be generalisable across countries, or to flag up where caution is needed in making generalisations.

If they were to be implemented on a population scale, risk-stratified breast screening programmes would have to adhere to gold standards of the Wilson and Jungner criteria [16] and additional criteria [17]. In particular, they should ensure that “all components of the screening program be clinically, socially and ethically acceptable to screening participants, health professionals and society” and that there are “effective methods for providing screening participants with informed choice, promoting their autonomy and protecting their rights” [18, 19]. In addition, specific challenges raised by risk stratification need to be addressed, such as the shift in the focus of screening. Indeed, from the practice of diagnosis that aims at detecting the presence of a tumour, it evolves in a broader programme that incorporates risk assessment of developing that cancer in the future [20, 21]. This shift might affect individuals (e.g., anxiety, misunderstanding) but also society (overestimation of cancer risk in the population, stigmatisation and discrimination according to level of risk or non-participation to the screening programme). With these issues in mind, a specific work package within the MyPeBS project aims to examine the ethical, psychological and socio-economic impact of the new screening proposition. This paper describes the research protocol of this work package, including justification of its specific objectives. Objectives and corresponding analytical tools are summarised in Appendix 2.

### Objectives

We aim to compare women in the risk-stratified arm and in the standard arm of MyPeBS, on the following issues: (1) anxiety and cancer worry; (2) understanding of breast cancer screening strategy and information seeking behaviour; (3) socio-demographic and economic characteristics and quality of life; (4) risk perception and intention to change behaviour; (5) acceptability and satisfaction of the trial.

1. The first objective of the research is to assess the emotional impact of risk communication on women. Amongst the potential harms of risk-stratified screening are undue increases in general anxiety or distress related to cancer. The results from a previous study assessing effects of breast cancer risk-stratification found little evidence for elevated distress, although risk information was communicated approximately three years after information on risk factors was elicited from participants [22]. The present study will assess if increased distress is a harm of risk-stratified breast cancer screening, by comparing, at one year, the changes from baseline in levels of general anxiety and cancer-specific worry between women in the risk-stratified arm and women in the standard arm.
2. The second objective aims to assess women’s comprehension of the information provided within MyPeBS, and their willingness to search for further information. One particular aim will be to test whether there is a correlation between women’s information comprehension level, information-seeking behaviours and their sociodemographic and socio-economic characteristics. As risk-based approaches rely on information and schedules that are more complex, it is essential that all decisions made in the context of screening programmes are informed and based on sound understanding of the information provided. Therefore, is it important to assess whether individuals have access to sufficient and clear information to decide to participate and to understand the proposed programme.
3. The third objective is to analyse MyPeBS participants' socio-economic and demographic characteristics as well as quality of life, to assess whether they are representative of the social heterogeneity of the participating countries, and whether these characteristics influence screening perceptions, uptake and behaviours, such as compliance with the proposed breast cancer screening programmes. This relates to a more general aim stated in previous research [23] to identify possible means to further universal access to screening programmes regardless of women’s socio-economic status, cultural origin or literacy level.
4. The fourth objective is to assess perception of risk of breast cancer, and perceptions of efficacy and risks of prevention options [24, 25]. Uptake of options that may reduce future risk of aggressive breast cancer, such as increased mammography frequency in women at high risk, healthier eating, or more physical activity, is linked to perceptions of risk of disease and of the benefits of these behaviours in reducing risk [11]. We will also assess to what extent perceptions of risks and benefits predict intentions to attend future screening and intentions to change lifestyle behaviours (healthy eating, physical activity), and whether they predict subsequent uptake of mammography.
5. The fifth objective aims at measuring overall satisfaction with the trial, and the acceptability of the risk-based approach, should it be implemented at a population scale. First, it is necessary to assess if women are satisfied with this new screening proposal, and with the organisation of the clinical trial, whether they are in the risk-stratified arm or in the standard arm. Furthermore, it is critical to collect women’s feedback on the information and communication materials that they receive throughout the trial, in order to anticipate future needs raised by the large-scale implementation of this new strategy.

In addition to the aforementioned issues which will be assessed globally and for each country, one further objective will be to compare the issues between countries. One of the strengths of the MyPeBS trial is the participation of six countries with specificities in terms of recruitment, local organisation of the trial and type of healthcare professionals involved. Adopting a comparative and contextualised perspective when analysing the results on acceptability of the trial, anxiety and cancer worry, understanding and information-seeking behaviours, equity, risk perception and intentions to change behaviour will offer a more nuanced view on the psychological and socio-economic issues at stake with risk-based approaches.

## Methods and design

### Design of the methodology

In order to meet the described objectives, this work package was designed with two complementary methodologies: a quantitative approach with questionnaires administered to each woman participating in MyPeBS, and a qualitative study consisting of 48 semi-structured interviews conducted with women randomised in the risk-stratified arm in France and in the UK. The use of qualitative methods, in addition to quantitative measures, is recommended in guidance on process evaluation [26], as the two kinds of methods have contrasting strengths.

The quantitative questionnaires will provide measures for the previously stated objectives, at various time points during the trial. Qualitative methods will help further investigate perceptions of risk-stratified screening and collect feedback on the trial from the participants. This qualitative work will give insight into how acceptable women found the process of being offered risk-stratified screening, and their experiences of participating in the trial.

The multi-disciplinary structure of the research team (comprised of researchers in psychology, sociology, health economics, and ethics together with epidemiologists, radiologists, geneticists, patients advocates and clinicians) enriched the design of the methodology of this study. The methodology was reviewed and validated by the project’s executive committee and then approved by ethics committees in each participating country. Data collection and processing are compliant with the European General Data Protection Regulation.

### Participants

Women from the general population (no prior breast cancer and not already identified at high risk) aged 40 to 70 are eligible if they live in a participating area from one of the six participating countries. Women are either invited to participate by the organised breast screening programme structures in each participating country or by a healthcare professional, or she can self-volunteer; indeed, information about the study was disseminated in healthcare providers’ premises (private offices, public screening centres, hospitals), in the media, and through health insurances’ newsletters. Candidates who are interested in participating in the trial and who are eligible with respect to the inclusion and exclusion criteria are registered and randomised via an online dedicated web platform (Eonix, Belgium), and receive a unique identification code that ensures anonymity of data treatment. The participants’ informed consent is obtained by the recruiter before any study-related procedures are performed. The first participant was included on 18^th^ July 2019 and inclusion is still ongoing.

For the qualitative substudy, participants who undergo risk-stratified screening in the MyPeBS trial are recruited by letters sent via screening centres in Manchester (UK) and in the regions of Paris and North of France (France). Study in the UK attempts to recruit a range of women from different levels of socio-economic status. Further informed consent is gained by the study researcher before participants take part in the qualitative study.

### Procedure

**Fig. 1:**
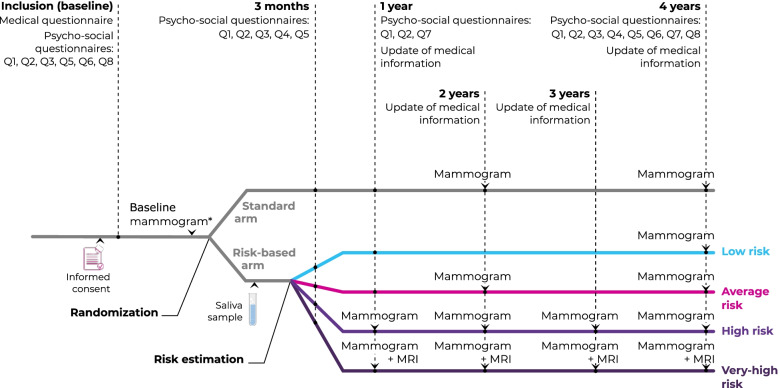
Design of MyPeBS and timing of questionnaire administration. *The baseline mammogram can be performed before or after randomization. If participants had a mammogram in the year prior to recruitment, they do not have to do new one. Psycho-social questionnaires: Q1: STAI (state of anxiety), Q2: Lerman cancer worry scale, Q3: Comprehension, Q4: Information-seeking behaviour and health literacy, Q5: Risk perception, Q6: Quality of life (EQ-5D), Q7: Satisfaction, Q8: Socio-demographic and economic status

Psychosocial questionnaires (see Fig. 1) are administered on the web platform to women at different times during the trial, namely at inclusion (baseline), at three months, at one year and at four years (end of trial). Women complete the baseline psycho-social and medical questionnaires before the randomisation process. Both steps are performed by the recruiter during the inclusion visit. For each other stage of the trial, women receive an invitation by email to complete the next questionnaires. Participants are given a one-month window and receive weekly email reminders to complete the three-months form online. At one year and four years, they are given a two-months window and bi-monthly reminders to fill in the forms. The study sponsor (Unicancer) is responsible for data management and ensures quality control, storage and exportation of data for dedicated analyses.

For the qualitative study, the research team will conduct 48 semi-structured interviews with women in the risk-stratified arm of the trial, both in France (*n* = 24) and in the UK (*n* = 24). The aim is to recruit six women per risk group (low, average, high and very high), in order to reach data saturation for each of those. Recruitment in France will be carried out with the help of Haut-de-France’s and Ile-de-France’s Regional centres for coordinating cancer screening (CRCDC). Recruitment in the UK will be carried out with help from the Manchester University NHS Foundation Trust (MFT). The interviews will provide an in-depth account of women’s views and experiences of the breast cancer screening programme and allow investigation of how healthcare systems and cultural differences affect acceptability of the proposition.

### Sample size justification

The present study is embedded within the main MyPeBS trial, which aims to recruit 85,000 women from six countries, in 2.5 years, and randomise them to receive either usual screening or risk-stratified screening. The sample size for the main MyPeBS study was based on providing sufficient statistical power to detect non-inferiority of risk-stratified screening in progression of participants to stage 2 breast cancer or beyond.

The present consideration of socio-psychological aspects of risk-stratified screening in the MyPeBS trial will involve the recruitment and follow-up of the entire study sample of women in the MyPeBS trial. This will provide very high statistical power for the five main objectives of the present study. For example, as of December 31st, 2021, 22,884 participants have been recruited, and assuming 70% follow-up of the first 22,000 women, the present study will have over 99% power to detect a very small difference (standard mean difference of 0.08) between the two experimental groups.^3^ Thus, the present sample size will provide more than sufficient statistical power for the five main objectives presented in this paper, and allow well-powered comparisons of these variables in each of the six countries (the sixth objective).

## Measures

### Quantitative questionnaires

Among the questionnaires selected, most are validated questionnaires previously used, some of them in different languages. Appropriate licences have been obtained.

Several questionnaires were created to fit this study aims: the comprehension questionnaire, the information seeking-behaviour questionnaire and the satisfaction questionnaires were built according to previously described methodologies [27–29]. When necessary, the questionnaires were translated to English, Italian, French, Spanish, Dutch, Arabic, Hebrew and Russian, which are the languages commonly used in the six countries of the trial. Translation and reverse translation to English were performed by an official sworn translator. The three versions (English, target language and back translation into English) were then proofread by bilingual professionals involved in the MyPeBS trial. A review of the online versions by the professionals involved in MyPeBS allowed for a final adjustment in the wording. The following subsections give more details on methodological aspects of these questionnaires.

#### State-Trait Anxiety Inventory (STAI) (Q1) and Lerman Cancer Worry scales (Q2) (at baseline, three months, one year and four years)

In order to assess if increased distress is a harm of risk-stratified breast cancer screening, the research team will compare the levels of general anxiety and cancer-specific worry between women who receive risk-stratified screening and women who receive standard screening. Cancer-specific worry measures seem to be more sensitive to the effects of receiving risk information than more general measures of anxiety [30, 31]. By contrast, there is evidence that more general measures of anxiety are better linked to diagnoses of psychological disorders [32]. For this reason, we assess both indices of psychological distress, using standardised measures, namely the short form State-Trait Anxiety Inventory (STAI) to assess general state anxiety [33] and the Lerman cancer-worry scale to assess cancer-specific worry [34].

The Lerman Cancer worry scale questionnaire has been used in similar studies evaluating cancer risk assessment and clinical programmes [22, 35]. The STAI short form questionnaire is a validated questionnaire which has been implemented across many studies, including breast cancer risk-stratification research [22]. Both scales have six items with four response options each. Composite scores are then calculated, with score ranges between 4 and 24, with higher scores indicating higher anxiety.

There is also evidence that both general anxiety and cancer-related worry peak shortly after receiving information on cancer risk and then gradually decline [30, 31]. For this reason, we will assess these variables over time and across the six countries, to examine whether there are short-term increases as well as any longer-term effects.

As levels of anxiety might be affected if women have their mammogram the day they fill in those questionnaires, information on whether or not a mammogram is planned on the day of inclusion will be collected.

#### Comprehension questionnaire (Q3) (at baseline, three months, and four years)

Patients' understanding of health information is a key issue in the exercise of their autonomy, and an ethical principle. This is why different texts—including legal, such as informed consent, or deontological, such as the code of medical ethics or the patient's charter—specify that the physician must provide the patient with a clear, fair and appropriate information on his or her health state, and on the investigations and care proposed. Providing information to the patient and ensuring that he or she understands is a necessary, even if not sufficient, condition for informed or shared decision-making to occur, and is a major public health issue, including in the case of breast cancer screening [36–38].

For all of these reasons, we decided to assess the comprehension of the information provided in MyPeBS. A comprehension questionnaire was developed based on our previous studies [27, 39] and on information tools used by investigators during the accrual visits. The questionnaire contains fifteen true/false questions divided into three categories: (i) general information on breast cancer; (ii) information on breast cancer risk; (iii) information on benefits and risks of breast cancer screening. As this questionnaire was developed for use in this specific study, the questionnaire was piloted amongst a set group of individuals: first, with three healthcare professionals to ensure the scientific relevance of the proposed questions, then with ten women aged 40 to 70. This pilot phase was carried out using face-to-face interviews based on an interview guide containing the main items (clarity of questions, length of questionnaire, redundant or missing questions, etc.) and also the "thinking aloud" procedure [40]. The content was analysed, and questionnaires were improved, on the basis of notes taken during these interviews and their recordings [41, 42].

The comprehension questionnaire will be administered to women at three months and four years. In order to test if responses are not due to pure chance, a Q Cochran test will be used to assess this.

#### Information-seeking behaviour questionnaire (Q4) (at three months, and four years)

Based on previous research conducted by our research team on French cancer survivors’ living conditions, we decided to explore women's information-seeking behaviours. This research showed that patients’ comprehension of information provided is related to their information-seeking behaviours [28]. Patients who did not understand information provided by healthcare professionals, as well as patients who did not obtain responses to their questions, were obliged to search for information on their own, especially in the case of patients with a low socioeconomic status who might perceive themselves as “dropouts” of the healthcare system. To investigate these information-seeking behaviours, we adapted the questionnaire that was developed in this previous research and added five questions from the Health Literacy Questionnaire (HLQ) focused on the “search for information” dimension [43], keeping in mind the previously described trend for an association between inadequate health literacy and lower breast cancer screening rates [44].

Our questionnaire asks whether women searched for information in the first place, their reasons for doing so, and the type of information they looked for (e.g., on the internet, in the media, or by asking healthcare professionals). Some questions also relate to breast cancer screening in general, and to risk-stratified breast cancer screening issues in particular.

As this questionnaire was developed for use in this specific study, the questions were piloted (with the exception of the five questions of the HLQ questionnaire already validated) amongst the same group of ten women and with the same methods as the ones used for the comprehension questionnaire.

The information-seeking behaviour questionnaire is administered at three months and four years to all participants of MyPeBS.

#### Risk perception and behaviour change (Q5) (at baseline, three months, and four years)

As previously underlined, it is essential that all decisions made as part of breast cancer screening are informed and based on sound understanding of good quality information provided. A key element of understanding is how accurately women perceive their own risk of breast cancer following risk-stratified screening, and whether this has been improved, relative to standard screening. For these reasons, we will assess perception of risk of breast cancer, and perceptions of efficacy and risks of different prevention options, to assess the constructs of Protection Motivation Theory, a theory that aims to understand how risk information can translate into behaviour change or not [45]. Uptake of options that may reduce future risk of breast cancer such as increased frequency of mammography in women at high risk, or increases in healthy eating or physical activity, is linked to perceptions of the risk of disease and benefits of these behaviours in reducing risk [11]. By contrast, a potential harm of risk-stratified screening is that those who are told they are low risk may increase their unhealthy behaviours through being falsely reassured (i.e., a certificate of health effect).

To estimate comparative risk and comparative severity scores, participants are asked to choose a category indicating their perceived risk of invasive breast cancer over the next ten years, compared to the population’s average (comparative risk). They are also asked to evaluate, if they were to get breast cancer, how severe they think this would be (comparative severity) on a five-point scale from lower to higher risk.

The behaviour change questions evaluate three different behaviours relating to reducing breast cancer risk: improving diet, increasing physical activity and attending mammography appointments. Five-point scales are used to measure Response Efficacy (the perception of how much the behaviour would reduce the participant’s risk of breast cancer) and Self-Efficacy (how easy or difficult it would be to enact these behaviours). To calculate the intention to change behaviour scores, participants are asked three questions assessing their intention to change the three health-related behaviours on a five-point scale, from “strongly disagree” to “strongly agree” [22].

#### Quality of life (Q6) (at baseline and four years)

To assess women’s quality of life, we use the EQ-5D licensed by EuroQoL research foundation. This European quality of life tool is a validated pre-scored multi-attribute health status scale that has been used in a wide range of populations and situations, including breast cancer screening [46–49]. It is short and easy to complete. It is divided into two parts: a first part with five items representing five dimensions, i.e. mobility, self-care, usual activities, pain and discomfort, anxiety and depression. The patient is asked to respond on a five-point scale: 1, no problem; 2, mild problems; 3, moderate problems; 4, severe problems; 5, extreme problems or total disability. A second part with a visual analog scale “EQ-5D VAS”, a 20-cm line graduated from 0 to 100 allows the patient to rate his or her current health status, with 0 being the worst health status and 100 the best.

The EQ-5D is administered at baseline and at four years to analyse and compare women’s quality of life between the two screening options, i.e. standard versus personalised breast cancer screening, and between groups of different risk levels. We will thus explore whether women who underwent risk-stratified screening have a better quality of life than women who underwent standard screening. Quality of life among different socio-economic subgroups will also be compared and related to breast cancer screening compliance, using both aggregated and unaggregated measures (i.e. considering the five dimensions of the EQ-5D separately) of quality of life, based on the responses to the EQ-5D questionnaire.

#### Trial Programme Satisfaction (Q7) (at one year and four years)

This questionnaire is an evaluation of the overall satisfaction of women with their participation in the study, with the information provided and communication tools used, and with the overall organisation of the trial. The intention to participate in the study is assessed at one year and actualised at four years. Analyses will consider the dropout rate. The questionnaire is an adapted version of a satisfaction questionnaire on organisation of the screening process and the information received, validated by Bairati et al. [29] and currently used in Canada. The questionnaire was modified to integrate specific questions concerning the MyPeBS study. It was validated along with the comprehension and information-seeking behaviour questionnaires, following the same methodology, and consists of seven questions with five-point scale answers.

#### Socio-demographic questionnaire (Q8) (at baseline and four years)

The characteristics of women participating in MyPeBS versus those taking part in the current population-based screening programmes will be analysed and compared, in terms of socio-demographics (age, education level, profession, income level, geographic area, marital status) and socio-economic status. In particular, we will focus on social inequalities by using the nine items version of the Material Deprivation Index (MDI), keeping in mind that socio-economic deprivation is a well-established determinant for low uptake and low compliance to cancer screenings [50] including breast cancer screening [51].

The MDI is an aggregated measure of deprivation validated in many European countries, based on the EU-SILC survey that has already been used to assess inequalities in cancer screening access [52]. This index has been the most used in the literature to assess material deprivation and make international comparisons. This index has several strengths: (i) the multi-dimensionality of the nine-items MDI has been psychometrically validated, (ii) it is theoretically justified, in particular because it makes the distinction between lack of items (due to choice) and enforced lack of items because of poor economic resources, (iii) validated threshold values are derived to classify individuals into “material deprivation” or “severe material deprivation”, (iv) the proportion of materially deprived individuals in twenty-seven European countries has been calculated based on the EU-SILC survey, thus allowing for comparison of representativeness of MyPeBS participants.

The MDI score is calculated by scoring 1 if the person answers “no, I cannot afford it” to the item and then by summing all items (thus the MDI ranges between 0 and 9). A threshold value of three of the MDI is used to classify individuals into “material deprivation” and a threshold of is used to classify individuals into “severe material deprivation”.

The impact of socio-demographics and deprivation levels on women's adherence to schedule during the trial will be analysed. Women’s socio-demographic and socio-economic characteristics will be analysed at baseline and four years and will be compared to the characteristics of women taking part in the current population-based screening.

### Qualitative interview guide

Participants will be asked about how acceptable they found the risk-stratified process, their understanding of the purpose of each phase, how they experienced receiving their risk category, if they would recommend this type of screening to their friends or family, and how the process could be improved. Demographic information to describe the sample of participants will be collected on the same occasion as the main interview. Interviews will last approximately one hour and will be conducted face-to-face, via telephone or video call, according to the Covid-19 sanitary situation and restriction measures.

With the consent of participants, interviews will be audio-recorded and transcribed in the languages in which they were conducted, using an accredited transcription company, to ensure data security. Interviews conducted in French will then be translated into English, since it is the language used for the analysis. All transcribed interviews will be checked by the research team to ensure accuracy and to remove information that could allow the identification of interviewees.

### Analyses

The quantitative analysis plan (see Appendix 1) includes: 1) descriptive statistics (description of categorical and quantitative variables, tests and estimation of confidence intervals); 2) bivariate analyses, subgroups comparisons and analysis of variations in time (baseline, three months, one year and four years), with appropriate tests to assess differences in groups (e.g. chi-square, Fisher and Student, Q Cochran); 3) multivariate regression models (cross-sectional and longitudinal) to model different patient reported outcomes such as comprehension of the information provided, general anxiety or cancer worry, and information-seeking behaviours. The data management and statistical analyses will be performed using Stata (StataCorp LP) and SPSS (IBM Corp). The main endpoint of this study will be measured at the end of the four years of intervention.

The qualitative data will support the interpretation of findings from the quantitative data. It will use framework analysis, a form of thematic analysis that facilitates the handling of large qualitative datasets [53]. It involves examining qualitative data to produce themes that summarise and interpret patterns of results. The analysis will take an explicit comparative approach, to investigate differences between England and France, and will also consider how the level of estimated risk affects reactions to risk-stratified screening and how acceptable the women found this. Coding will continue until the team is satisfied that codes and themes adequately describe and capture the data and that saturation has been achieved. Data will be stored and organised within Nvivo software (QSR International Pty Ltd. Version 12, 2018).

Dissemination of the study results will include the publication of research papers. A more direct feedback to investigators and to participants is planned at various stages of the trial.

## Discussion and conclusion

As of December 31^st^, 2021, 22,884 participants have been included and randomised in the trial and have completed baseline questionnaires. Inclusion for the qualitative study started in November 2021 in France and will start in March 2022 in the UK.

The Covid crisis resulted in a six-months suspension of trial enrolment as of March 2020, and still has a major detrimental impact on the recruitment, as successive epidemic waves continue to hinder both women and healthcare teams to participate in the study. Furthermore, when the enrolment reopened, one of the priorities was to reduce participants' time on site as much as possible. It was therefore decided to have women complete the first two questionnaires (STAI and Lerman cancer worry scale) at the inclusion site prior to randomisation, while the other questionnaires could be completed afterwards, either at the inclusion site or at home. This allowed the scientific requirements for assessing anxiety levels to be maintained prior to the start of the study, while adapting the conditions of inclusion to the health context.

One point to note is that the French Ethics committee limited the range of questionnaires proposed to women in France, due to concerns around heightened anxiety after answering the STAI-6 (Q1) and Lerman cancer worry scale (Q2). These questionnaires, however, will be administered in the five other countries taking part in the trial.

Along with investigating women’s perceptions of risk-based approaches and the socio-economic issues raised by this new form of screening, feedback is also needed on professionals’ perceptions of the MyPeBS trial and more generally on risk-based approaches. There is a dearth of research on clinicians’ views on implementing such approaches [54]. With this in mind, a complementary quantitative substudy is currently investigating professionals’ perceptions and attitudes regarding the risk-based breast cancer screening approach, the possible difficulties with giving information to women, delivering risk categories or announcing a change in the frequency of screening. The substudy will collect feedback on MyPeBS, on the training and communication tools provided to professionals by the study sponsor, on their overall satisfaction and on their specific difficulties encountered during recruitment and follow-up of participating women. If the risk-based strategy were to be implemented at a population level, having basic feedback from professionals on communication and training tools would be valuable. Moreover, getting information on their understanding of risk categories and genotyping will help specify their needs for further training.

Beyond the scientific and medical objectives of this clinical study, it is critical to pay attention to the consequences of such a paradigm shift for women on the one hand and for professionals on the other. Indeed, introducing a risk-based screening relying on individual biological differences also implies acknowledging non-biological differences (e.g. social status and health literacy) from an ethical perspective to ensure equal access to healthcare [21, 55]. Thus, through different methodologies and multidisciplinary work, this study aims to evaluate the potential psychological effects for women when their risk of developing breast cancer is assessed. It also aims to evaluate information needs, based on the evaluation of understanding of information transmitted at the time of entry into the study, and when they receive their risk category and their personalised screening schedule. These results will contribute to issuing of recommendations at the end of the trial to accompany any potential change in screening strategy at the European level, even though they should be adapted to specific local contexts. The description of the socio-economic categories of women who participated will also allow us to assess whether the study reached women of all socio-economic levels and whether interventions specifically dedicated to socio-economic categories with less participation should be put in place before generalisation.

### Supplementary Information

**Additional file 1.** N°1 list of Ethical Committees involved in MyPeBS.

**Additional file 2. **N°2 list of study sites involved in MyPeBS.

## Data Availability

Not applicable.

## Appendix 1

### Statistical analysis plan

#### a. Software used

Data management and statistical analyses will be performed using Stata (StataCorp) and SPSS (IBM Corp).

#### b. Descriptive statistics

The categorical variables will be described by the numbers and percentages observed on each modality. Missing values will not be included in the calculation of percentages. The number of missing values will be indicated. In order to assess the uncertainty around the estimation, 95% confidence intervals according to exact binomial distribution will be estimated for the parameters of interest.

The quantitative variables will be described by the usual statistics: mean, standard deviation, median, minimum, maximum, first and third quartile. The other percentiles will be provided if necessary.

#### c. Bivariate analyses and subgroups comparisons

The bivariate analyses will mainly focus on detecting differences between subgroups (e.g., countries). Before, it will be ensured that each group contains a sufficient number of participants and is adequately powered.

For the qualitative or discrete variables, the significant differences between subgroups will be assessed either by means of the Chi-square test or by Fisher's exact test depending on the conditions of statistical validity (i.e., theoretical number strictly less than 5).

The Cochran Mantel Haentszel test (or Q Cochran) will test for equality of proportions in matched samples. It will be used to test, for instance, how the comprehension level of risk stratified screening varies in time, (i.e., between baseline, three months and four years).

For quantitative variables, significant differences between two subgroups will be detected via Analysis of Covariance (ANCOVA), controlling for baseline levels of those variables, and any other clinical or demographic variables that are not well balanced between subgroups, and hence may have a confounding effect. Initial assumption testing will include the Fischer test, which will be used to verify the hypothesis of equality of variances, and the Shapiro Wilk test, which will be used to verify the hypothesis of normality. In the case of non-normality, non-parametric approaches will be used in sensitivity analyses. In this case, the Wilcoxon or Mann–Whitney test for comparisons between two subgroups and the Kruskal–Wallis test beyond two subgroups will be considered.

The tests will be bilateral with a significance level of 5%. Adjustments for multiple comparisons (Bonferroni correction) do not appear to be required.

According to the literature, no method for managing missing data is unanimously accepted. As a result, automatic replacement of missing data will not be used [56]. The analyses will be conducted on all patients included with no missing data. In case of no answer, we will assume that the data are missing at random. This assumption will be tested by comparing the characteristics of respondents and non-respondents using the Chi-square statistic. Another source of missing data will likely come from women not answering subsequent waves of the survey (for instance, questionnaires at one year, two years or four years). In other words, we may face an attrition issue. First, we will test whether attrition is non exogenous (i.e., data are not missing at random), which could bias our estimates from longitudinal models assessing the evolution of our main dependent variables over time. In order to test for exogenous attrition, we will compare the results from the balanced (i.e., with no missing data) and unbalanced (i.e., with missing data points) panel using an Hausman test [57]. Rejection of the null assumption of equality of coefficients between the two panels will provide evidence of non-exogenous attrition. In case non exogenous attrition is detected, we will use inverse probability weighting to correct for the selection effect, i.e., we will weight women by the inverse of their probability of selection [58]. This method has been shown to reduce the selection bias in epidemiological applications.

#### d. Multivariate regression analyses

Multivariate regression models will be used to model different patient reported outcomes (PROs) such as comprehension of the information provided, general anxiety or cancer worry, information seeking behaviour, etc. Both cross-sectional and longitudinal models will be estimated.

For all models, a threshold of 5% will be used to assess statistical significance of model parameters.

##### d.1. Cross-sectional models

Logit models will be used to estimate binary dependent variables models. For categorical (ordered) dependent variables, the proportionality of odds-ratio conditions will first be assessed to test the suitability of estimating ordered logit models. If the condition is not verified, multinomial logit models will be estimated. For continuous dependent variables, linear models will be considered. Normality and homoscedasticity of error terms will first be tested before estimation. In case of rejection of normality, generalised linear models (GLMs) will be estimated and in case of rejection of homoscedasticity, heteroscedastic-consistent standard errors will be computed.

Regressors of interests will be selected based on the literature, results of qualitative pre-tests and a priori assumptions. In case many regressors are selected, stepwise selection methods will be considered in order to estimate the most parsimonious model.

##### d.2. Longitudinal models

In order to analyse the changes of an outcome variable with time, longitudinal (or panel) data models will be estimated. The estimated models belong to the family of generalised linear mixed models (GLMMs). The link function and distribution of error terms will be defined depending on the nature of the dependent variable. In the GLMMs models, only the intercept will be specified as random in order to capture repetition of data across individuals. It will also be possible to account for various levels of heterogeneity across countries by including country specific random effects (multilevel model). Time-fixed effects will be included in the model to assess changes of a dependent variable over time, and interactions between time-fixed effects and regressors of interest will be included to test for changes in the impact of a variable over time. Potential attrition bias will be assessed and accounted for using the methods described above (missing data management).

## Appendix 2

Table

**Table 1:** Summary of objectives, statistical analyses and tools

Objective	Tools	Timing	Analysis
Assess the psychological impact of risk communication on women	- STAI (Q1) - Lerman’s cancer worry (Q2) - Qualitative interviews	Baseline, 3 months, 1 year, 4 years	- Comparisons between two experimental arms, controlling for baseline levels and other covariates where required
Assess women’s comprehension of the information provided within MyPeBS, and their willingness to search for further information	- Comprehension questionnaire (Q3) - Information seeking behaviours questionnaire (Q4) - Qualitative interviews	Baseline, 3 months, 4 years	- Comparisons between arms and correlations between women’s information comprehension level, information-seeking behaviours and socio economic and demographic characteristics - Correlations with psychological characteristics (anxiety, cancer worry)
Analyse MyPeBS participants' socio-economic and demographic characteristics as well as quality of life	- Quality of life (Q6) - Socio-demographic questionnaire (Q8)	Baseline, 4 years	- Comparisons between arms of women’s socio-economic characteristics and quality of life, focus on health inequalities. Use equality of proportion or Student test to compare the distribution of the variables across the two harms - Correlations with risk perceptions and screening uptake
Assess perception of risk of breast cancer, and perceptions of efficacy and risks of prevention options	- Risk perception and behaviour change (Q5) - Information-seeking behaviour questionnaire (Q4) - Qualitative interviews	Baseline, 3 months, 4 years	- Comparisons between two experimental arms, controlling for baseline levels and other covariates where required - Examination of mediation effects on intentions and behaviour by risk appraisals and coping appraisals
Measure overall satisfaction with the trial and acceptability of the risk-based approach	- Trial Program Satisfaction (Q7) - Qualitative interviews	1 year, 4 years	- Comparison between arms and analysis of critical points within experimental arm

1

## Abbreviations

ANCOVA: Analysis of Covariance
CRCDC: French regional centres for coordinating cancer screening
EQ-5D: European quality of life validated tool
GLMs: Generalised linear models
GLMMS: Generalised linear mixed models
HLQ: Health Literacy Questionnaire
MDI: Material Deprivation Index
MyPeBS: “My Personal Breast Screening”
PROs: Patient reported outcomes
STAI: State-Trait Anxiety Inventory

## Footnotes

1 WCRF International. World cancer research fund international. 2012. Available from: http://www.wcrf.org/int/cancer-facts-figures/data-specific-cancers/breast-cancer-statistics.

2 *MyPeBS—International Randomized Study Comparing personalized, Risk-Stratified to Standard Breast Cancer Screening In Women Aged 40-70—*https://www.mypebs.eu/the-project/—coordinated by UNICANCER as sponsor.

3 In relation to the first objective concerning effects on anxiety (see section below), a standard mean difference of 0.08 is equivalent to a difference between adjacent response categories (e.g. “not at all” and “somewhat”) on just one of the 20 items on the full form of the scale, assuming the mean anxiety score of 37 and standard deviation of 12 that has been found in the control group of a previous study with women in England invited to breast cancer screening (22).
