# Supplementary material for: Study protocol comparing the ethical, psychological and socio-economic impact of personalised breast cancer screening to that of standard screening in the “My Personal Breast Screening” (MyPeBS) randomised clinical trial
Source: BMC Cancer. 2022 May 6;22:507. doi: 10.1186/s12885-022-09484-6 (PMC9073478; doi:10.1186/s12885-022-09484-6)
Supplement: Supplementary file 1 — Additional file 1. N°1 list of Ethical Committees involved in MyPeBS. [file 12885_2022_9484_MOESM1_ESM.docx]

**Additional File N°1 list of Ethical Committees involved in MyPeBS**

| Pays | Type | Nom |
| --- | --- | --- |
| BEL | Central | Institut Jules Bordet |
|  | Local | CHU Brugmann |
|  | Local | CHIREC DELTA |
|  | Local | CHU St Pierre |
|  | Local | Hopitaux Iris Sud - HIS Ixelles |
|  | Local | UZBrussel |
|  | Local | Centre Hospitalier de Wallonie - Picardie |
|  | Local | UZLeuven |
|  | Local | Clinique Universitaire St luc |
|  | Local | Centre de Sénologie des Drs Crèveceour |
|  | Local | CH JOLIMENT |
|  | Local | Centre de Sénologie des Drs Crèveceour |
| ESP | Central | Comité de Ética de la Investigación con medicamentos del Parc de Salut Mar |
| FRA | Central | CPP Sud Ouest et Outre Mer 4 |
| GBR | Central | London - Chelsea REC |
| ISR | Central | Helsinki committee |
| ITA | Central | CE dell'Area Vasta Emilia Nord |
|  | Local | CEROM Romagna |
|  | Local | CE Interaziendale - Torino |
|  | Local | CER Toscana - AREA VASTA CENTRO |
|  | Local | CERC - Veneto |
|  | Local | CE Ospedale San Raffaele |
|  | Local | CE di Bergamo |
|  | Local | CE Regionale delle Marche |
|  | Local | CER Toscana - AREA VASTA SUD EST |
|  | Local | CEUR - Trieste |
